# Supplementary material for: Seroprevalence and Associated Risk Factors of Bovine Brucellosis at the Wildlife-Livestock-Human Interface in Rwanda
Source: Microorganisms. 2020 Oct 9;8(10):1553. doi: 10.3390/microorganisms8101553 (PMC7600169; doi:10.3390/microorganisms8101553)
Supplement: Supplementary file 1 [file microorganisms-08-01553-s001.zip › Table S1.docx]

Table S1: Univariate associations of potential risk factors with herd-level seropositivity of *Brucella* spp in cattle sampled at the wildlife - livestock - human interface in Rwanda.

| **Variables** | **Category** | **Interviewed** | **RBT & i-ELISA** | | ***p-*value** |
| --- | --- | --- | --- | --- | --- |
|  |  |  | **No. (%)** | **[95% CI]** |  |
| Level of education | Tertiary-secondary | 44 | 4 (9.09) | [0.60 – 17.58] | 0.002^a^ |
|  | Primary | 83 | 25 (30.12) | [20.25 - 39.99] |  |
|  | No education | 85 | 32 (37.65) | [27.35 - 47.95] |  |
| Herd size | Small ≤ 10 | 72 | 20 (27.78) | [17.43 - 38.12] | 0.33 |
|  | Medium ≤ 11 ≤ 30 | 116 | 31 (26.72) | [18.67 - 34.78] |  |
|  | Large ≥ 31 | 24 | 10 (41.67) | [21.94 - 61.39] |  |
| Herd composition | Cattle only | 108 | 22 (20.37) | [12.78 - 27.97] | 0.007^a^ |
|  | Cattle-SR* | 44 | 20 (45.45) | [30.74 - 60.17] |  |
|  | Cattle-dog | 60 | 19 (31.67) | [19.9 – 43.44] |  |
| Grazing system | Zero grazing | 73 | 10 (13.7) | [5.81 – 21.59] | < 0.001^a^ |
|  | Free grazing | 139 | 51 (36.69) | [28.68 – 44.7] |  |
| Proximity to wildlife | Yes | 65 | 24 (36.92) | [25.19 - 48.65] | 0.072 |
|  | No | 147 | 37 (25.17) | [18.15 - 32.19] |  |
| Endemic diseases in the region | Brucellosis | 44 | 20 (45.45) | [30.74 - 60.17] | 0.022^a^ |
|  | HP diseases* | 62 | 17 (27.42) | [16.32 - 38.52] |  |
|  | Viral diseases* | 81 | 21 (25.93) | [16.38 - 35.47] |  |
|  | Mastitis | 25 | 3 (12) | [0.0 – 24.74] |  |
| Sharing watering points | Yes | 150 | 50 (33.33) | [25.79 - 40.88] | 0.034^a^ |
|  | No | 62 | 11 (17.74) | [8.23 – 27.25] |  |
| Fenced farms | Yes | 114 | 30 (26.32) | [18.23 – 34.4] | 0.48 |
|  | No | 98 | 31 (31.63) | [22.48 - 40.84] |  |
| History of infertility | Yes | 115 | 36 (31.30) | [22.83 - 39.78] | 0.46 |
|  | No | 97 | 25 (25.77) | [17.07 - 34.48] |  |
| History of abortions | Yes | 88 | 37 (42.05) | [31.73 - 52.36] | < 0.001^a^ |
|  | No | 124 | 23 (19.35) | [11.71 - 25.39] |  |
| Knowledge of brucellosis | Yes | 167 | 57 (34.13) | [26.94 - 41.32] | < 0.001^a^ |
|  | No | 45 | 4 (8.89) | [0.57 – 17.2] |  |
| Vaccination last two years | Yes | 26 | 6 (23.08) | [6.88 – 39.27] | 0.64 |
|  | No | 186 | 55 (29.57) | [23.01 - 36.13] |  |
| Breeding system | Natural | 192 | 57 (29.69) | [52.04 - 71.88] | 0.44 |
|  | AI | 20 | 4 (20) | [2.47 – 37.53] |  |
| Having own bull | Yes | 78 | 22 (28.21) | [18.22 - 38.19] | 0.832 |
|  | No | 114 | 35 (20.70) | [22.24 - 39.17] |  |
| Access to veterinary services | Yes | 105 | 23 (21.90) | [24.32 - 42.35] | 0.042^a^ |
|  | No | 107 | 38 (35.51) | [26.45 - 44.58] |  |
| Regular testing | Yes | 39 | 8 (20.51) | [7.84 – 33.18] | 0.29 |
|  | No | 173 | 53 (30.64) | [23.77 – 37.5] |  |
| Disinfection of abortion site & pastures | Yes | 3 | 0 (0) | [0.0 – 0.0] | 0.56 |
|  | No | 209 | 61 (29.19) | [23.02 - 35.35] |  |
| Introduction of new cattle | Yes | 115 | 41 (35.65) | [26.9 – 44.41] | 0.024^a^ |
|  | No | 97 | 20 (20.62) | [12.57 - 28.67] |  |
| Screening before introduction | Yes | 2 | 0 (0) | [0.0 – 0.0] | 0.54 |
|  | No | 113 | 41 (36.28) | [27.42 - 45.15] |  |
| Feeding abortive tissues to dogs | Yes | 101 | 42 (41.58) | [31.97 – 51.2] | < 0.001^a^ |
|  | No | 111 | 19 (17.12) | [10.11 - 24.12] |  |

No.: number of responses from owners of seropositive cattle

AI: artificial insemination

Cattle-SR: cattle and small ruminants

HP diseases: Hemoparasitic diseases (Theileriosis, Trypanosomiasis)

VD: Viral diseases (Foot and mouth disease)

*^a^p* < 0.05: proportions are significantly different.
