# Supplementary material for: Seroprevalence and Associated Risk Factors of Bovine Brucellosis at the Wildlife-Livestock-Human Interface in Rwanda
Source: Microorganisms. 2020 Oct 9;8(10):1553. doi: 10.3390/microorganisms8101553 (PMC7600169; doi:10.3390/microorganisms8101553)
Supplement: Supplementary file 1 [file microorganisms-08-01553-s001.zip › Questionnaire .pdf]

QUESTIONNAIRE FOR FARMERS, FARM WORKERS AND VETERINARIANS

This questionnaire (interview) is designed to collect information about the prevalence and risk factors of brucellosis and abortions in cattle and goats. These data will be used to inform policy makers and service providers to improve monitoring and surveillance of infectious diseases. This interview will take 5 min. The owner's names, farm's name, and names of farm workers will be kept confidential.

This questionnaire contains 4 sections: information about the interview, respondent address and information, herd management, and knowledge of the diseases by respondents.

I. Information about the interview

1.1. Interview language:---
1.2. Interviewer name: ---

	**Day/month/year**
Date of first interview attempt	
Date and time arranged for second interview attempt	
Date and time arranged for third interview attempt	
Date of interview	
Date form checked by supervisor	
Date entered in computer	

II. Respondent information

2.1. Sectors of origin

Rwimiyaga ◯ Karangazi ◯ Bigogwe ◯ Kinigi ◯ Ndera ◯

2.2. Cell and village of origin / GPS

Cell ………………………..Village……………………GPS………………………………

2.3. Sex of respondents: Female ◯ Male ◯

2.4. Are you? Herd owner ◯ farm worker ◯ sector veterinarian ◯ district veterinarian ◯

2.5. How long have you been in this job < 1 year ◯ 2 - 3 years ◯ 4-5 years ◯ > 5 years ◯

2.6. Education level: Tertiary education ◯ Secondary education ◯ Primary ◯ None ◯

III. HERD MANAGEMENT

3.1. How many cattle do you have on this farm/sector/district? Less than 10 ◯ Between 11-20 ◯

Between 21-30 ◯ between 31 – 40 ◯ between 41 – 50 ◯ Above 50 ◯ ……………

3.2. What are the species present on this farm? Cattle ◯ Goats ◯ Sheep ◯ Dog ◯

3.3. What is the purpose of cattle production?

Meat ◯ Milk ◯ Mixed ◯ Prestige ◯

3.4. What is the market of milk produced?

Own consumption ◯ Inyange industries ◯ cooperatives ◯ others ◯

If others which ones?

3.5. Milking procedures: manual ◯ milking machine ◯

3.6. Do you boil your milk before selling or before home consumption? Yes ◯ No ◯

3.7. Do you make cream milk at home? Yes ◯ No ◯

3.8. Do you make butter, cow oil from milk at home? Yes ◯ No ◯

3.9. If your answer is yes, do you boil milk before? Yes ◯ No ◯

3.10. Do you use cow oil at home? Yes ◯ No ◯

3.11. If yes do you mix with food or fly the food? Mix ◯ fly ◯

3.12. Is there any cohabitation between wildlife and domestic animals? Yes ◯ No ◯

If yes go to question 3.13 if no go to question 3.14

3.13. Which kind of wildlife animals do you always see in contact with domestic animals?

Buffalos ◯ zebra ◯ gazelles ◯ elephants ◯ others ◯

If others please mention them

3.14. How often do you see wildlife animals?

Regularly ◯ sometimes ◯ rarely ◯

3.15. In which season or month do you see wildlife animals living the park?

Heavy sunny (June – September) ◯ light rainy (October – December) ◯

light sunny (January – February) ◯ heavy rainy season (March – may) ◯ regularly ◯

3.16. Do you share water sources with others? Shared ◯ personal ◯

3.17. What is the source of water? REG ◯ rain ◯ river ◯ other ◯

If others please mention ……………………………………………………………………………..

IV. KNOWLEDGE OF THE DISEASES

4.1. Are you aware of the transmission of contagious diseases from wildlife to domestic animals?

Yes ◯ No ◯

4.2. If yes which of the following diseases do you often find in your animals?

Tuberculosis ◯ Brucellosis ◯ Typanosomiasis ◯ Anaplasmosis ◯ Easter cost fever ◯

If others which ones: …………………………………………………………………………………

4.3. Do know brucellosis? Yes ◯ no ◯

4.4. Did you ever face infertility cases in your herd? Yes ◯ no ◯

4.5. Can brucellosis affect humans? Yes ◯ no ◯

4.6. Did you ever get sick of brucellosis or disease similar to Malaria? Yes ◯ no ◯

4.7. How often does calving occur? Naturally ◯ assisted ◯ c – section ◯

4.8. If assisted do you wear protective clothes during assisted calving? Yes ◯ no ◯

4.9. Did you ever see some abortions in your herd? Yes ◯ no ◯

4.10. How do you dispose the abortive organs? Dogs ◯ deep burial ◯ open air ◯

4.11. Do you vaccinate against brucellosis? Yes ◯ No ◯

If yes when last did you vaccinate against brucellosis? Every year ◯ this year ◯ last year ◯

4.12. Do you consult veterinarians? Yes ◯ no ◯

4.13. Do veterinarians come regularly to collect samples? Yes ◯ no ◯

If yes which ones?

Blood ◯ faeces ◯ foetuses ◯ abortion tissues ◯

4.14. What type of reproduction do you use? Natural mating ◯ artificial insemination ◯

4.15. Do you have your own bull? Yes ◯ no ◯

4.16. Do you know other diseases that are characterized by abortions? ◯ Yes ◯ no

If yes which ones? ……………………………………………………………………………

3.14. Do you clean & disinfect animals, stall, and pasture? Yes ◯ no ◯

3.15. If yes how many times a month? once ◯ twice ◯ more than twice ◯

3.16. What chemical do you usually use for cleaning & disinfection? …………………………………

3.17. Where did animals come from? In country ◯ Uganda ◯ Tanzania ◯

3.18. Did you perform any test before their entry? Yes ◯ No ◯

If yes which one? Rose Bengal test ◯ if others please specify……………………………………

3.19. Do you sometimes see animals crossing borders? Yes ◯ No ◯

If yes by which means of transport? By vehicle ◯ by feet ◯

Note: Questions highlighted in Gray are related to public health

IKINYARWANDA

IBIBAZO BIZIFASHISHWA MU KIGANIRO N' ABOROZI, ABAKOZI, N'ABAVUZI Z' AMATUNGO

Ibi bibazo bizifashishwa mu gushakisha amakuru ajyanye n'indwara y'amakore n' ukuramburura mu nka n' ihene. Aya makuru azafasha abashinzwe ubuhinzi n'ubworozi gukaza ingamba zo gukumira indwara zandura zifata abantu, amatungo, n'inyamaswa. Iki kiganiro kizamara iminota itanu (5). Amazina yanyu cyangwa y' ifamu azagirwa ibanga.

Ibi bibazo bigabanyijemo ibice bine (4): gahunda y' ikiganiro, umwirondoro w'ubazwa, imicungire y'ubushyo, n' ubumenyi ku ndwara z'amatungo n'abantu.

1. Gahunda

1.1. Ururimi ruzakoreshwa: --
1.2. Izina ry' uzayobara ikiganiro:--

	**Umunsi / ukwezi/umwaka**
Itariki ya mbere y'ikiganiro	
Itariki n'isaha by' ikiganiro ku inshuro ya 2	
Itariki n'isaha by' ikiganiro ku inshuro ya 3	
Itariki n'isaha by' ikiganiro nyirizina	
Itariki ibi bibazo byasuzumwe na mwarimu	
Itariki ibibazo n'ibisubizo byinjiriye muri mudasobwa	

2. Umwirondoro w'ubazwa

2.1. Umurenge ukomokamo

Rwimiyaga ◯ Karangazi ◯ Bigogwe ◯ Kinigi ◯ Ndera ◯

2.2. Icyo akora: Umworozi ◯ Umukozi ◯ Umuveterineri w'Umurenge ◯ w'Akarere ◯

2.3. Akagari, Umudugudu, aho aherereye (GPS)

Akagari ……………………..umudugugu...……………………GPS………………………………

2.4. Igistina : Gore ◯ Gabo ◯

2.5. Umaze igihe kingana iki muri aka kazi? umwaka 1 ◯ imyaka 2 - 3 ◯ imyaka 4 ◯

2.6. Wize ayahe mashuri? Kaminuza ◯ Ayisumbuye ◯ Abanza ◯ Nta mashuri ◯

3. **IMICUNGIRE Y' UBUSHYO**

3.1. Mufite inka zingahe muri ubu bworozi / Umurenge / Akarere? Munsi y'icumi 10 ◯ Hagati 11-20 ◯

Hagati 21-30 ◯ Hagati 31 – 40 ◯ hagati 41 – 50 ◯ Hejuru ya 50 ◯ ……………..

3.2. Ni ayahe matungo mufite hano? Inka ◯ ihene ◯ intama ◯ imbwa ◯

3.3. Intego yanyu ni ukorora inka zitanga amata cyangwa umukamo?

Inyama ◯ Amata ◯ byombi ◯ icyubahiro ◯

3.4. Niba ari umukamo, amata muyagurisha he?

Kwinywera mu rugo ◯ Inyange ◯ Koperative ◯ abandi ◯

Niba ari abandi ni bande?

3.5. Uburyo bwifashishwa mu gukama: amaboko ◯ imashini ikama ◯

3.6. Mubanza guteka amata mbere yo kuyagurisha cyangwa kuyanywa? Yego ◯ oya ◯

3.7. Mujya mukore ikivuguto mu rugo? Yego ◯ oya ◯

3.8. Mujya mukora amavuta y' inka mu rugo? Yego ◯ oya ◯

3.9. Niba mukora ikivuguto cyangwa amavuta mujya mubanza kubiza amata? Yego ◯ oya ◯

3.10. Mujya murya amavuta y'inka? Yego ◯ oya ◯

3.11. Niba ari yego murayarunga cyangwa murayakarangisha? kurunga ◯ gukaranga ◯ byombi ◯

3.12. Mujya mubona amatungo arisha ari kumwe n'inyamaswa? Yego ◯ Oya ◯

Niba ari yego komeza ku kibazo cya 8 niba ari oya komeza ku cya 9

3.13. Ni izihe nyamaswa mukunda kubona ziri hamwe n' amatungo?

Imbogo ◯ imparage ◯ gazelle ◯ inzovu ◯ izindi ◯

Niba hari izindi wazivuga

3.14. Izo nyamaswa uzibona kangahe?

Buri munsi ◯ rimwe na rimwe ◯ gake cyane ◯

3.15. Mu kihe gihe cyangwa ukwezi mukunze kubona inyamaswa ziva muri pariki?

impeshyi (Kamena – Nzeli) ◯ Umuhindo (Ukwakira – Ukuboza) ◯

Urugaryi (Mutarama – Gashyantare) ◯ Itumba (Werurwe – Gicurasi) ◯ buri gihe ◯

3.16. Amazi y'inka (ibumbiro) hari abo murifatanyije? Yego ◯ Oya ◯

3.17. Ayo mazi aturuka he? REG ◯ imvura ◯ umugezi ◯ ahandi ◯

4. UBUMENYI KU NDWARA Z' AMATUNGO

4.1. Muzi ko indwara z'inyamaswa zafata n' amatungo? Yego ◯ Oya ◯

4.2. Ni izihe ndwara mu kunze kubona mu bworozi?

Igituntu ◯ amakore ◯ indwara y'ibitotsi ◯ Gasheshe ◯ Ikibagarira ◯

niba hari izindi ni izihe? …………………………………………………………………………

4.3. Muzi amakore? Yego ◯ Oya ◯

4.4. Hari inka zanze kwima inshuro nyinshi? Yego ◯ Oya ◯

4.5. Ese abantu barwara amakore? Yego ◯ Oya ◯

4.6. Waba warigeze kurwara amakore cyangwa indi ndwara imeze nka Malariya? Yego ◯ Oya ◯

4.7. Ese hari inka zigeze kuramburura? Yego ◯ Oya ◯

4.8. Ibirambu bishyira he? Imbwa ◯ kubitaba ◯ turabyihorera ◯

4.9. Ese inka zikunze kubyara gute? Ku bwazo ◯ zifashijwe ◯ zibazwe ◯

4.10. Iyo muzifasha kubyara muba mwambaye akarindantoki cyangwa itaburiya? Yego ◯ oya ◯

4.11. Mujya mukingira amakore? Yego ◯ Oya ◯

Niba ari yego uheruka gukingira ryari? Buri mwaka ◯ uyu mwaka ◯ umwaka ushize ◯

4.12. Mujya mwitabaza abavuzi b' amatungo? Yego ◯ Oya ◯

4.13. Abavuzi b'amatungo bajya baza gufata ibizami? Yego ◯ Oya ◯

Niba ari yego bafata ibihe bizami?

Amaraso ◯ amase ◯ ibirambu ◯ uturemangingo tw'ibirambu ◯

4.14. Ni ubuhe buryo mukoresha mubangurira? Ikimasa ◯ Gutera intanga ◯

4.15. Ufite ikimasa cyawe? Yego ◯ Oya ◯

◯

4.16. Hari izindi ndwara muzi zitera inka kuramburura? Yego Oya

Niba ari yego ni izihe? ……………………………………………………………………………
4.17. Mujya musukura ikiraro, ubwatsi n' amatungo mukoresheje umuti? Yego Oya

Niba ari yego ni kangahe mu kwezi? Rimwe Kabiri birenze kabiri

4.18. Mukoresha uwuhe muti? ……………………………………………………………

4.19. Mugurira hehe inka? Mu gihugu mu Bugande muri Tanzaniya

4.20. Hari ikizamini wafashe mbere yo kugura? Yego Oya

Niba ari yego ni ibihe? Amaraso niba hari ibindi ni ibihe……………………………………
4.21. Mujya mubona inka zambukiranya imipaka? Yego Oya

Niba ari yego zambuka gute? Ziri mu modoka n' amaguru

<u>ICYITONDERWA:</u> IBIBAZO BIRI MU IBARA RY'IKIGINA BIJYANYE N'IBIKORWA BISHOBORA GUTUMA ABANTU BANDURA AMAKORE Y'AMATUNGO CYANGWA IMISUHA.
